# Glycated Hemoglobin Differences Among Blog-Reading Adults With Type 1 Diabetes Compared With Those Who Do Not Read Blogs: Cross-Sectional Study

**DOI:** 10.2196/13634

**Published:** 2019-04-02

**Authors:** Sean M Oser, Heather L Stuckey, Jessica A Parascando, Erin L McGinley, Arthur Berg, Tamara K Oser

**Affiliations:** 1 Department of Family and Community Medicine Penn State College of Medicine Hershey, PA United States; 2 Department of Internal Medicine Penn State College of Medicine Hershey, PA United States; 3 Department of Public Health Sciences Penn State College of Medicine Hershey, PA United States

**Keywords:** type 1 diabetes, blogs, continuous glucose monitor, insulin therapy, HbA_1c_, social media

## Abstract

**Background:**

Of the estimated 23.1 million individuals diagnosed with diabetes, approximately 5% have type 1 diabetes (T1D). It has been proposed that this number will triple by 2050. With increases in technology use and resources available, many individuals are using insulin pumps and continuous glucose monitors (CGMs) to help manage their T1D. They are also using online resources such as social media to find more information and advice based on real-life experiences from peers. Blogs are a particular social media modality often used by people with T1D but have not been widely investigated.

**Objective:**

The purpose of this study was to assess glycated hemoglobin (HbA_1c_) differences between blog readers and blog nonusers in a population of adults with T1D. This study also looked at differences in technology use in these two groups, as well as HbA_1c_ differences in blog use and technology subgroups.

**Methods:**

Participants were recruited both by mail and by online T1D-themed blog postings. Respondents completed a secure online eligibility assessment and were asked questions related to their T1D, blog and internet use, and insulin pump and CGM use. Demographics were also collected. Differences between blog readers and blog nonusers were tested via chi-square and *t* tests. Mann-Whitney *U* tests, Fisher exact tests, and analyses of variance (ANOVA) were used to test for differences in self-reported HbA_1c_ between groups and subgroups.

**Results:**

A total of 282 eligible participants completed the survey (214 blog readers, 68 blog nonusers). Average duration of diabetes was 21.2 years, 77.7% (219/282) were female, 81.2% (229/282) used an insulin pump, 66.3% (187/282) used a CGM, and 95.7% (270/282) were white. HbA_1c_ was lower for blog readers (7.0%) than blog nonusers (7.5%), *P*=.006; for insulin pump users (7.0%) than multiple daily injections (7.7%), *P*=.001; and for CGM users (7.0%) than CGM nonusers (7.5%), *P*=.001. After adjusting for significant covariates, the association between blog use and HbA_1c_ remained significant (*P*=.04). ANOVA modeling also demonstrated significant differences in HbA_1c_ between blog users and nonusers among subgroups by pump use and CGM use (*P*<.001).

**Conclusions:**

These results suggest that reading blogs is associated with lower HbA_1c_ values. While association does not prove causation, blog readers have the benefit of learning information from peers and having 24/7 access to a community of individuals with similar daily life struggles, where they are able to ask questions and seek advice.

## Introduction

Approximately 1.25 million persons in the United States have been diagnosed with type 1 diabetes (T1D) [1], with incidence increasing in recent years [2]. It is estimated that annual and lifetime costs are greater for individuals with T1D than for individuals with type 2 diabetes, as complications associated with T1D are more dire and often involve more intensive medical care [3]. The complications associated with dysregulation of blood glucose levels can lead to long-term and devastating health consequences. While it has been shown that maintaining blood glucose levels as close to normal as possible is associated with better health outcomes [4], most people with T1D do not achieve recommended blood glucose targets [5,6].

In efforts to combat fluctuating blood glucose and glycated hemoglobin (HbA_1c_) levels and reduce the incidence and severity of complications, T1D management has been facilitated by the introduction and evolution of diabetes technology such as self-monitoring of blood glucose and, more recently, continuous glucose monitors (CGMs) and increasingly automated insulin pumps.

Self-monitoring of blood glucose has demonstrated its fundamental importance: higher frequency of daily checks is correlated with lower HbA_1c_ [7,8]. Likewise, the use of an insulin pump has also demonstrated benefit in improving HbA_1c_ and decreasing risk of severe hypoglycemia and diabetic ketoacidosis [9], but pumps remain in use by a minority of patients [10,11,12]. As CGMs have evolved for patient use, earlier mixed results [13,14,15] have shifted to show benefits [16,17], especially when using CGM to augment insulin pump therapy [18-21].

In addition to these advances, social media has become a technology-based tool for health self-management for numerous conditions [22-29]. The Diabetes Online Community (DOC) has emerged as a popular, self-vetted community for caregivers of and persons with T1D, facilitating discussion of management strategies and personal experiences [30]. Specifically, the DOC provides patients and their caregivers with peer support in managing their T1D [31,32]. Data on social media use in diabetes continues to emerge. Health benefits have been shown when patients interact with their health team via social media [33]. Positivity and perceived benefit have been demonstrated among T1D patients connecting via social networking sites such as Instagram [34], Twitter [35], and Facebook [36]. Many have sought diabetes-related health information from social media platforms such as YouTube, Twitter, and Facebook [37,38,39] as well as from blogs [40,41,42]. And while there are limited data on an association between glycemia and social media engagement [43], no studies to date have examined a potential relationship between glycemia and blog use specifically.

Blogs allow extended and asynchronous sharing of personal experiences and reflections. They are available publicly and allow other readers to comment, or simply to read without commenting, when and where convenient for them. Blogs provide a particularly efficient source for retrospective analysis of data produced in a natural setting, gradually and without prompting. The methodology of blog analysis provides a foundational, time-efficient advantage over coordinating, conducting, and transcribing individual and/or focus group interviews.

The blogging community is broadly comprised of three types of users. Bloggers are those individuals who journal their experiences for others. Commenters read and actively comment on others’ posts. Lurkers read others’ blogs without commenting and are thought to comprise the largest of the three groups [44]. This study focused on lurkers and sought to determine whether there might be an association between HbA_1c_ and reading T1D-themed blogs. This paper describes key differences between HbA_1c_ and technology use among blog readers and blog nonusers.

## Methods

### Study Approval

This study used an online survey of adults with T1D and was approved by the Penn State College of Medicine Institutional Review Board. Implied consent was obtained online prior to the user being directed to the secure online survey. A summary explanation of the research served as implied consent and described the survey purpose and procedures, length of time to complete the survey, data storage and protection plans, and study team contact information, including name and phone number of the principal investigator. It was stated in the consent that the survey was voluntary.

### Recruitment

In efforts to reach a broad and varied sample of participants, recruitment was conducted via online announcement on two popular adult T1D blogs, as well as through mailing to all people over 18 years old and listed as having T1D in a diabetes registry hosted at an academic medical center*.* Both online and via mail, a link to the online survey was provided; following the link to the survey was entirely voluntary. Inclusion criteria were (1) at least 18 years of age, (2) a diagnosis of T1D, and (3) agreement to participate. Active writers of T1D blogs or active commenters on such blogs were excluded (by self-identifying as such in an eligibility question), as the focus of this study was on lurkers who read but do not contribute to blog content. The number of responses was based on a convenience sample, as this was an open survey, available to an unlimited number of persons who had access to the blogs and social media platforms where the survey link was available for two weeks. Duplicate complete responses were not allowed from the same IP address, as automatic IP detection by the online survey platform prevented this, although it did allow an incomplete response to be restarted until a complete response was received. Participants were not compensated for completion of the survey.

### Survey Instrument

Survey questions and results were housed in REDCap [45], a secure survey management research tool, at the Penn State Clinical and Translational Science Institute. Responses were automatically captured in REDCap and given a unique, sequential participant number. REDCap also captured date and time of survey responses, as well as completeness or partial completeness of surveys. Participants were asked inclusion and exclusion questions at the start of the survey, and access to the full survey was conditional upon meeting the aforementioned inclusion criteria. The survey included items assessing T1D-related blog use, insulin pump use, CGM use, internet access, mobile phone use, self-reported HbA_1c_, and demographics. The questions were tested among a subset of adults with T1D, refined, and retested until they were clearly understood and unambiguous. It was estimated that survey completion would take 5 to 10 minutes. Participants were not able to go back to the survey and edit their responses.

### Statistical Analysis

Statistical analysis was conducted using SPSS Statistics version 24 (IBM Corp). Chi-square tests and *t* tests, as appropriate, were used to test for differences in participant characteristics between blog readers and blog nonusers. Differences in self-reported HbA_1c_ between blog readers and blog nonusers were tested using the Mann-Whitney *U* test and Fisher exact test for continuous and categorical data, respectively. Analysis of variance (ANOVA) was used to identify statistical associations of HbA_1c_ levels across various subgroups of the survey participants.

## Results

### Recruitment

Response to online recruitment efforts was brisk, with the first response coming within minutes of the opportunity being posted on one blog. There were 65 responses within the first 24 hours of that posting and 185 within 24 hours of posting to a second blog (48 hours after the first one). Responses to the letters mailed to registry patients were fairly brisk as well, with 30 responses within 3 days of the letters being mailed. There was a slowdown over the Thanksgiving holiday, when there was no mail delivery, totaling 41 responses through the first week, and then response continued at a steady pace of about 15 per week until the survey closed two weeks later, with 70 responses to the mailed letters.

### Sample Demographics

Of the 472 people who began the survey, 74 did not meet the inclusion criteria, and 116 did not complete the survey. Of the 282 who completed the survey (59.7% [282/472] of those who started the survey, 70.9% [282/398] of those eligible), 214 were blog readers and 68 were blog nonusers. Table 1 shows characteristics of the entire sample and divided by blog use/nonuse. Mean duration of diabetes was 21.2 [SD 13.7] years, 77.7% (219/282) were female, 81.2% (229/282) used an insulin pump, 66.3% (187/282) used a CGM, and 75.9% (214/282) were blog readers; 97.9% (276/282) were non-Hispanic, 95.7% (270/282) were white, and 75.9% (214/282) were employed. Comparing blog readers to blog nonusers, there were significant differences between the two groups in age, gender, employment, insulin pump use, CGM use, marital status, and mobile phone use, with blog readers more likely to be under age 45, female, and employed and more likely to use an insulin pump, CGM, and mobile phone. Blog nonusers were more evenly split by gender and had a higher proportion of students and retirees than the sample of blog readers. In both groups, the most common marital status was married or domestic partnership, but blog readers were slightly more likely to be single/never married, and blog nonusers were slightly more likely to be divorced or separated. There were no significant differences between the groups in education, ethnicity, race, time since diagnosis, or method of internet access. Income information was surveyed but is not reported due to an especially large proportion of respondents who declined to answer this question in particular.

### Hemoglobin A_1c_ Associations

HbA_1c_ was significantly lower for blog readers than for blog nonusers (7.0% vs 7.5%, *P*=.006). After adjustment for significant covariates, this difference retained significance (*P*=.04). HbA_1c_ was also significantly lower for insulin pump users than for multiple daily injections (7.0% vs 7.7%, *P*=.001) and for CGM users than for CGM nonusers (7.0% vs 7.5%, *P*=.001). Other than these three variables (blog reading, pump use, CGM use), no significant differences in HbA_1c_ were found across any of the other variables collected.

**Figure 1:**
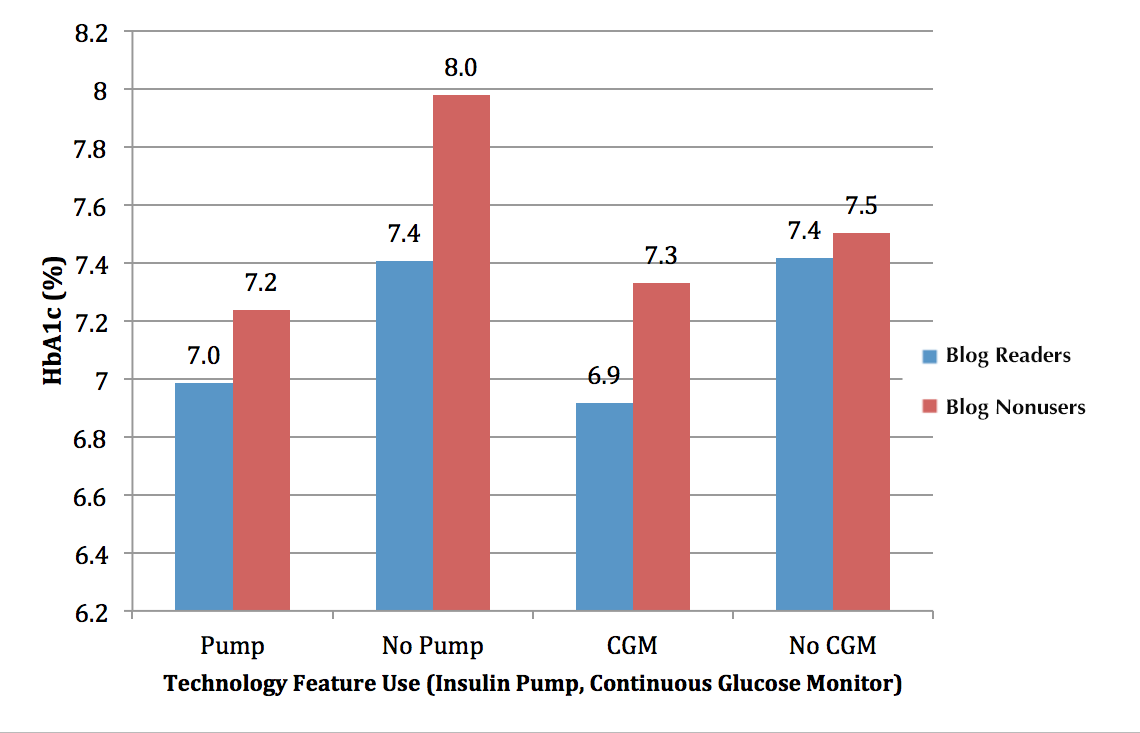
Hemoglobin A_1c_ (HbA_1c_) among blog readers and blog nonusers, by insulin pump and continuous glucose monitor (CGM) use.

To evaluate HbA_1c_ by blog use and a second independent variable (eg, blog use and insulin pump use), respondents were divided into four groups across the two variables (eg, blog readers on insulin pump, blog readers not on insulin pump, blog nonusers on insulin pump, blog nonusers not on insulin pump). Similar subgroups were also constructed with the binary variables blog use and CGM use. Differences in HbA_1c_ were seen across these groups as well, as depicted in Figure 1. HbA_1c_ was lowest among blog readers who use an insulin pump (7.0%) and highest among blog nonusers who do not use an insulin pump (8.0%); intermediate levels were seen among insulin pump users who do not read blogs (7.2%) and blog readers who do not use an insulin pump (7.4%). ANOVA evaluation of HbA_1c_ by insulin pump use and blog use revealed a strong statistical association between blog/insulin pump use and HbA_1c_ (*P*<.001). In addition to this strong association, there was no significant interaction between blog use and pump use (*P*=.22).

Two-factor ANOVA also showed differences in HbA_1c_ by blog use and CGM use, which is also demonstrated in Figure 1. HbA_1c_ was lowest among blog readers who use a CGM (6.9%) and highest among blog nonusers who do not use a CGM (7.5%); intermediate levels were seen among CGM users who do not read blogs (7.4%) and blog readers who do not use a CGM (7.4%). ANOVA evaluation of HbA_1c_ by blog/CGM revealed a strongly significant statistical association with HbA_1c_ (*P*<.001). Additionally, there was no statistically significant interaction between blog use and CGM use (*P*=.24).

**Table 1:** Sample description by blog use group.

Characteristics		Total sample (n=282)	Blog readers (n=214)	Blog nonusers (n=68)	*P* value
**Age (years), n (%)**					.009^a^
	18-24	27 (9.6)	20 (9.3)	7 (10.3)	
	25-34	94 (33.3)	80 (37.4)	14 (20.6)	
	35-44	73 (25.9)	57 (26.6)	16 (23.5)	
	45-54	42 (14.9)	25 (11.7)	17 (25.0)	
	55-64	29 (10.3)	24 (11.2)	5 (7.4)	
	65-74	16 (5.7)	8 (3.7)	8 (11.8)	
	75+	1 (0.4)	0 (0)	1 (1.5)	
**Gender, n (%)**					<.001^a^
	Male	62 (22.0)	34 (15.9)	39 (57.4)	
	Female	219 (77.7)	180 (84.1)	28 (41.2)	
	Unspecified	1 (0.4)	0 (0)	1 (1.5)	
**Education level, n (%)**					0.12
	High school graduate	11 (3.9)	5 (2.3)	6 (8.8)	
	Some college	53 (18.8)	37 (17.3)	16 (23.5)	
	College graduate	104 (36.9)	82 (38.3)	22 (32.4)	
	Postgraduate degree	114 (40.4)	90 (42.1)	24 (35.3)	
Ethnicity: Hispanic, n (%)		6 (2.1)	5 (2.3)	1 (1.5)	0.64
**Race, n (%)**					0.49
	Asian	5 (1.8)	4 (1.9)	1 (1.5)	
	Black or African American	6 (2.1)	3 (1.4)	3 (4.4)	
	Native American	1 (0.4)	1 (0.5)	0 (0)	
	White	270 (95.7)	206 (96.3)	64 (94.1)	
**Employment, n (%)**					<.001^a^
	Employed	214 (75.9)	175 (81.8)	39 (57.4)	
	Student	21 (7.4)	14 (6.5)	7 (10.3)	
	Retired	18 (6.4)	6 (2.8)	12 (17.6)	
Years since diagnosis, mean (SD)		21.2 (13.7)	20.3 (12.9)	24.0 (15.7)	0.13
Insulin pump use, n (%)		229 (81.2)	181 (84.6)	48 (70.6)	.009^a^
CGM^b^ use, n (%)		187 (66.3)	156 (72.9)	31 (45.6)	<.001^a^
**Marital status, n (%)**					.045^a^
	Single, never married	73 (25.9)	58 (27.1)	15 (22.1)	
	Married/domestic partnership	182 (64.5)	141 (65.9)	41 (60.3)	
	Separated/divorced	23 (8.2)	13 (6.1)	10 (14.7)	
**Internet access method, n (%)**					0.52
	Desktop	69 (24.6)	48 (22.4)	21 (31.3)	
	Laptop	70 (24.9)	58 (27.1)	12 (17.9)	
	Tablet	35 (12.5)	26 (12.1)	9 (13.4)	
	Mobile phone	107 (38.1)	82 (38.3)	25 (37.3)	
Mobile phone use, n (%)		258 (91.5)	202 (94.4)	56 (82.4)	.004^a^

^a^Significant at the *P*<.05 level.

^b^CGM: continuous glucose monitor.

**Table 2:** Analysis of variance of hemoglobin A_1c_ by blog use status and a second variable.

Second variable	*P* value			
	Model	Blog use	2nd variable	Interaction
Pump use^a^	<.001^b^	.012^b^	.001^b^	.22^c^
Continuous glucose monitor use^a^	<.001^b^	.04^b^	.03^b^	.24^c^
Marital status	<.001^b^	.45	.006^b^	.20^c^
Employment	.001^b^	.58	.25	.10^c^
Age	.002^b^	.09	.11	.66^c^
Education	.007^b^	.04^b^	.19	.09^c^
Gender	.008^b^	.001^b^	.23	.17^c^
Time since diagnosis	.02^b^	.01^b^	.10	.07^c^
Ethnicity	.03^b^	.37	.20	.83^c^
Smartphone use	.04^b^	.11	.37	.75^c^
Race	.01^b^	.40	.40	.03
Internet access method	.15	.03^b^	.95	.44^c^

^a^No significant interaction; significance achieved for ANOVA model and additive effects of blog use and second variable.

^b^Significant at the *P*<.05 level.

^c^No interaction of the two test variables (*P*>.05).

Analysis of HbA_1c_ by blog use and other variables is summarized in Table 2. This table provides *P* values associated with the overall effect of the two variables (blog use and the second variable), the additive effects associated with blog use and the second variable, and the interaction effects of the two variables. For example, in ANOVA modeling of HbA_1c_ by blog use and age, the ANOVA model was significant (*P*=.002) and there was no significant interaction (*P*=.66) but neither variable had a significant individual additive effect (*P*=.09, *P*=.11).

## Discussion

### Principal Findings

In this study, blog use—but not interacting with insulin pump use or CGM use—was found to be a predictor of HbA_1c_. To the best of our knowledge, this is the first study to evaluate blog use and HbA_1c_ and, therefore, the first to find an association between these two variables.

HbA_1c_ was 0.5% lower among blog readers than among those who don’t read blogs. Beside being statistically significant, this is a clinically significant difference approaching the magnitude seen in this study with CGM use (0.5%) and insulin pump use (0.6%). The HbA_1c_ differences among pump users and CGM users in this study are similar to associations found by others, which are also acknowledged to be clinically important [6,9,46-49]. Likewise, the HbA_1c_ difference seen here between blog readers and blog nonusers may have significant clinical implications as well.

This raises the question of why this was seen, as it is not possible to determine any causal relationship here. It could be that more technologically inclined individuals are more likely to use insulin pumps and/or CGMs than those less technologically inclined, and it would therefore stand to reason that they would likewise be more likely to go online (including to read blogs and to respond to an online survey to determine study eligibility). It could also relate to insurance, financial, and/or policy differences that make some more and some less likely to have access to technology. These could be areas for future research—for example, to investigate whether there is a true clinical benefit to blog use or other internet-based technology use, as appears may be the case in this study’s technologically inclined sample.

It is also possible that the results may be understood in the context of dynamic social impact theory, which posits that communication can more effectively increase an individual’s likelihood of changing behavior if (1) the communicator is credible and similar to the reader, (2) the communication is temporally immediate, and (3) there are multiple persuasive change agents communicating [50]. In the context of reading blogs, it may be that as individuals living with T1D observe the experiences described by peers on readily available blogs, which are reinforced by commenters, they are more likely to change behaviors related to T1D self-management, resulting in improvements in HbA_1c_. Further research is needed to better understand the role blogs may play in T1D self-management.

### Limitations

A limitation of this study is that HbA_1c_ values here are self-reported; although this is more likely to have an effect on the absolute values themselves than on the magnitude of the differences observed, and self-report has been shown to be highly reliable elsewhere [51]. Another limitation is the inability to determine and report response rate—it is impossible to know how many people viewed the online announcement, as it was spread via social media. However, the ability to recruit significant numbers of respondents online was remarkable, as was the speed with which they responded—an emerging phenomenon [52]. In some contrast, the letters mailed to individuals from the diabetes registry produced a good response, but it was more gradual, over the course of a few weeks. This resulted in a disparity in sample size between the two groups, as those recruited online outnumbered those recruited by direct mail. Despite this, both sample populations in this study demonstrate a substantially higher proportion of insulin pump use and CGM use than has been reported for the general population of patients with T1D in the United States [10,11,12]. Finally, when considering the HbA_1c_ differences between blog readers and blog nonusers, it should be noted that there were also demographic differences between the sample populations, for example, in age and employment status. The blog nonusers were more likely to be students or retired than blog readers, for example; although the implications of this are not clear. Blog readers were also more likely to be female, which is consistent with general statistics about blog users but does represent a difference between the two groups. However, even with these demographic differences, there were no significant associations between HbA_1c_ and any of these demographic factors.

### Future Directions

Other factors to study in the future might include potential differences in activation levels among blog readers compared to blog nonusers, as has been suggested elsewhere [53,54], as well as differences in levels of social support and/or instrumental support among blog readers compared to nonusers, in efforts to identify the reasons for the observed differences between adults with T1D who do and do not read T1D-themed blogs. As the social media landscape changes quickly and as blogs change in their popularity relative to other social media formats, a broader examination of potential relationships of HbA_1c_ to other types of social media could be informative. Other areas for future study could include replicating the study for adults with type 2 diabetes or for parents of children with T1D and examining how lurkers interact with blog content and the impact of these interactions on behaviors.

### Conclusions

This study found that blog readers had lower HbA_1c_ than blog nonusers. One possible explanation for these results is that access to blogs provides valuable information and experiences related to T1D. We also found that blog readers who used insulin pumps or CGMs had lower HbA_1c_ compared to blog nonusers who did not use insulin pumps or CGMs, suggesting that being technologically inclined, or having access to technology, is beneficial for T1D health outcomes. Clinicians may wish to consider recommending blogs or specific blog posts to their patients with diabetes if they are comfortable with the content.

## Abbreviations

ANOVA: analysis of variance
CGM: continuous glucose monitor
DOC: Diabetes Online Community
HbA_1c_: glycated hemoglobin
T1D: type 1 diabetes
